# New convolutional neural network model for screening and diagnosis of mammograms

**DOI:** 10.1371/journal.pone.0237674

**Published:** 2020-08-13

**Authors:** Chen Zhang, Jumin Zhao, Jing Niu, Dengao Li

**Affiliations:** 1 College of Information and Computer, Taiyuan University of Technology, Taiyuan, Shanxi, China; 2 Technology Research Center of Spatial Information Network Engineering of Shanxi, Taiyuan, Shanxi, China; 3 College of Data Science, Taiyuan University of Technology, Taiyuan, Shanxi, China; University of Craiova, ROMANIA

## Abstract

Breast cancer is the most common cancer in women and poses a great threat to women's life and health. Mammography is an effective method for the diagnosis of breast cancer, but the results are largely limited by the clinical experience of radiologists. Therefore, the main purpose of this study is to perform two-stage classification (Normal/Abnormal and Benign/Malignancy) of two- view mammograms through convolutional neural network. In this study, we constructed a multi-view feature fusion network model for classification of mammograms from two views, and we proposed a multi-scale attention DenseNet as the backbone network for feature extraction. The model consists of two independent branches, which are used to extract the features of two mammograms from different views. Our work mainly focuses on the construction of multi-scale convolution module and attention module. The final experimental results show that the model has achieved good performance in both classification tasks. We used the DDSM database to evaluate the proposed method. The accuracy, sensitivity and AUC values of normal and abnormal mammograms classification were 94.92%, 96.52% and 94.72%, respectively. And the accuracy, sensitivity and AUC values of benign and malignant mammograms classification were 95.24%, 96.11% and 95.03%, respectively.

## 1 Introduction

Breast cancer is the cancer with the highest incidence in women and is also an important cause of death. According to the prediction of the American Cancer Society, the number of new cases of breast cancer in women will reach 276480 in 2020, accounting for about 30% of new cancer cases in women [1]. And statistics show that since 2004, new cases of breast cancer have maintained an annual growth rate of about 0.3% [2], which seriously threatens women's life and health. However, studies have shown that if patients can be diagnosed with breast cancer in the early stage, their 5-year survival rate can reach 90%, which can be improved by 70% compare with the terminal stage [3]. Therefore, early diagnosis of breast cancer is of great significance to women's health.

Mammography has the characteristics of clear imaging and high resolution, and is an important means of breast cancer examination. Generally, radiologists need to make diagnosis according to the various characteristics of mammograms, combined with rich clinical experience. However, the diagnosis results could be limited by factors such as experience, visual fatigue, and image clarity. Therefore, a reliable computer-aided diagnosis (CAD) system is needed to help radiologists make a correct diagnosis. Related research has shown that the CAD system can effectively improve the diagnosis efficiency, reduces the rate of misdiagnosis and the burden of patients [4, 5]. In many medical institutions, CAD systems has been used in clinical diagnosis as doctors’ reference [6].

Traditional CAD methods usually need to manually extract features from images [7]. These features include original features such as shape and texture [8, 9], and the features extracted from the original features by machine learning algorithms, such as Histogram of Gradient [10–12], Local Binary Patter [13, 14] and Gabor filter [11, 12]. However, the selection and combination of features largely depend on the experience of designers, so the traditional methods have some limitations. The selection and combination of features depend largely on the experience of designers, so the traditional methods have some limitations.

In recent years, the research of deep learning has made great progress. Convolutional neural network (CNN) is an important branch of deep learning, which is widely used in the field of natural image analysis. Inspired by this, some researchers try to apply CNN to the field of medical image analysis [15–17] to help radiologists make correct judgments. In order to verify the effectiveness of CNN model in clinical diagnosis, Dontchos et al. [18] introduced external validation to evaluate the performance of their proposed model. They used the model to classify breast density and provided the classification results to radiologists for reference. With the assistance of this CNN model, the radiologists can significantly improve the accuracy of diagnosis.

For radiologists, it is usually necessary to observe the whole mammogram to achieve lesion screening, density classification and risk prediction. Therefore, some researchers have tried to apply the CNN model to the classification of the whole mammogram. Agnes et al. [19] constructed a new multi-scale fully convolution CNN model for the classification of normal, benign and malignant mammograms. Compared with single-scale filter, the multi-scale filter they used can extract a wider range of information. Mohamed et al. [20] used CNN to classify breast density, and they evaluated the classification accuracy of mammograms from two different views. This research can help different radiologists make consistent judgement. Charan et al. [21] used the CNN model to classify normal and abnormal mammograms. They segmented the breast area through morphological operations, which can effectively improve the classification performance of the model. Arefan et al. [22] verified the feasibility of using normal mammograms to predict the risk of breast cancer. They used GoogLeNet to extract the deep features of mammograms and classified them through linear discriminant analysis algorithm.

However, compare with the input size of the CNN model, the size of the whole mammogram is larger. Therefore, for the diagnosis of breast lesions, it is difficult to achieve good results by using the whole mammogram as the input of the CNN model. So some researchers have tried to use smaller image patches to classify the lesions. Khan et al. [23] used four image patches from the left and right breasts to classify the mammograms in three stages. The first stage is to divide the images into normal and abnormal, the second stage is to divide the abnormal images into mass and calcification, and the third stage is to divide the lesions into benign and malignant. Arora et al. [24] built an integrated neural network model to divide image patches into benign and malignant. The model integrated five popular neural network models, and connected the features extracted from these five networks through the fully connected layer, and output the final classification result. Chougrad et al. [25] constructed a deep convolutional neural network for diagnosing mammograms. They used three different network models in the experiment, and the Inception model achieved the best result. Although the use of image patches can improve the efficiency of feature extraction to a certain extent, it also ignores some global information related to diagnosis. At the same time, the diagnosis of image patches cannot help radiologists find abnormalities in a large number of mammograms.

In addition, the CNN model contains a large number of parameters. In order to achieve good classification performance, it often needs to use a large amount of data for training. However, in practical applications, it is usually difficult to obtain a large amount of labeled data, and transfer learning is an important method to overcome this difficulty. Yousaf et al. [26] used transfer learning to fine-tune nine CNN models pre-trained by ImageNet to achieve age-invariant face recognition. Wang et al. [27] used CNN to classify benign and malignant mammograms, and proved that the use of transfer learning on similar data can help improve the performance of the model. And the research of Matthews et al. [28] also proved this view. They used the full-field digital mammography database to train the proposed model, and generalized the model to the classification of digital breast tomosynthesis images through transfer learning. Samala et al. [29] used two CNN models to classify benign and malignant mammograms. In experiments, they verified that fixing the parameters of some convolution layers in the process of transfer learning can help to improve the classification performance and enhance the robustness of the model.

In this paper, in order to screen and diagnose of mammograms, we constructed a multi-view feature fusion network based on multi-scale attention DenseNet, and classified mammograms into two stages. Our contributions can be summarized as follows:

1. The same breast contains two mammograms from two views of craniocaudal (CC) and mediolateral oblique (MLO). The multi-view feature fusion network we proposed contains two independent CNN branches. These two CNN branches are used to extract the features of the mammograms from two views, respectively.
2. In order to extract the features of the whole mammograms more effectively, We constructed a new multi-scale convolution module and integrated it in DenseNet. The model uses filters with different scales to extract image features, so that the model can focus on the global and local features.
3. We proposed a new attention module, which connects channel attention module and spatial attention module in parallel. And We added this module to the multi-scale convolution module to improve the feature extraction ability.

## 2 Materials and methods

### 2.1 Materials

The Digital Database for Screening Mammography (DDSM) [30] is used to train and evaluate our proposed model, which is currently the largest publicly available database of mammography. Each case in the database consists of 4 mammograms from CC and MLO views of bilateral breasts, so a total of 10480 mammograms of 2620 cases are included. The database divides all cases into three categories: normal, benign and malignant. For each abnormal mammogram, some relevant diagnostic information including accurate benign and malignant diagnosis results, lesion type and breast density are provided.

#### 2.1.1 Data division and preprocessing

According to the diagnosis information of radiologists, we extract 1318 normal breasts, 753 benign breasts and 783 abnormal breasts from the database. Each of these breasts contains two mammograms from the CC and MLO views, so we obtain a total of 5706 mammograms of 2853 breasts. We divide all data into training set and testing set. The training set includes 1190 normal breasts and 1407 abnormal breasts (688 benign and 719 malignant). The testing set includes 128 normal breasts and 128 abnormal breasts (64 benign and 64 malignant). We divide the training set into 10 subsets in proportion, so that we can evaluate the performance of the model through ten-fold cross-validation. Table 1 shows the distribution of the data we used.

**Table 1:** Data distribution.

Type	Normal	Abnormal		Total
		Benign	Malignancy	
**Train**	1190	688	719	2597
**Test**	128	64	64	256
**Total**	1318	752	783	2853

Because of the high noise and poor contrast of original mammograms, it is often unable to achieve good classification performance when it is directly used as the input of convolutional neural network. Therefore, we need to perform some well-known preprocessing steps on the original mammograms, including contrast enhancement, bilateral filtering, and image normalization.

#### 2.1.2 Data enhancement

CNN model contains a lot of parameters, which often require a lot of data for training. If there is too little data in the training set, it may cause the model to overfit during the training, and reduce the generalization performance of the model. Therefore, in this study, in order to reduce the impact of overfitting, data enhancement technology is used to expand the training data. The single-view mammograms are first enhanced, and then the two-view mammograms are enhanced. It should be noted that these two steps are independent of each other, and both are expanded on the original data and used in different experiments.

(1) Data enhancement of single-view mammograms

In the process of data enhancement of single-view mammograms, we randomly combine the data enhancement strategies in the following two steps to expand the data by 50 times. First, the original mammograms are expanded by flipping up and down, flipping horizontally, randomly cropping, randomly scaling between 0.8 and 1 times, and translating by 10% in four directions. Then, the previously expanded mammograms are enhanced by adjusting the contrast, adding Gaussian noise, and randomly rotating between 0–180°.

(2) Data enhancement of two-view mammograms

There is a correlation between the mammograms from the two views. In order to avoid destroying the relationship, we only consider to expand the two-view mammograms properly by flipping. When the mammograms from CC view are flipped up and down, the mammograms from MLO view remain unchanged. When the mammograms from MLO view are flipped left and right, the mammograms from CC view are also flipped left and right.

### 2.2 Methods

**Fig 1:**
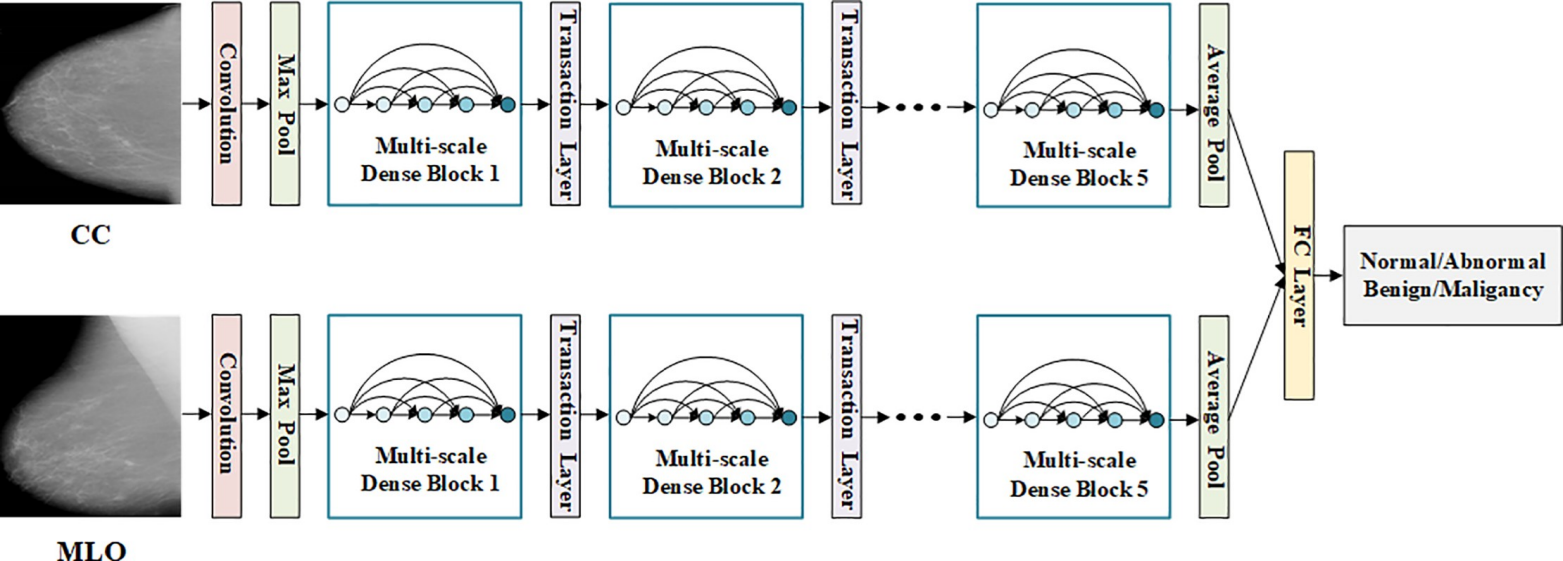
The overall structure of the MVNN model. Two multi-scale attention DenseNets are used to extract the features of mammograms from two views, then a fully connected layer is used to fuse these features, finally realize the classification of mammograms in two stages.

In this study, we proposed a multi-view feature fusion neural network model, and we named it as MVNN. The model takes mammograms from CC and MLO views as input, and realizes the classification of mammograms in two stages (Normal/Abnormal and Benign/Malignant). In the process of building the model, our work mainly focused on the following three aspects. First, we constructed a new attention module, which connects the channel attention and the spatial attention in parallel, and introduces the residual structure inside the module. Second, in order to extract the features of the whole mammogram, we introduced multi-scale convolution into Dense Block and constructed a multi-scale attention DenseNet. Third, we used two identical multi-scale attentional DenseNet to extract the features of mammograms from CC and MLO views respectively, and connected these features through a fully connected layer to realize the classification of mammograms. Fig 1 shows the overall structure of the MVNN model.

#### 2.2.1 Attention module

**Fig 2:**
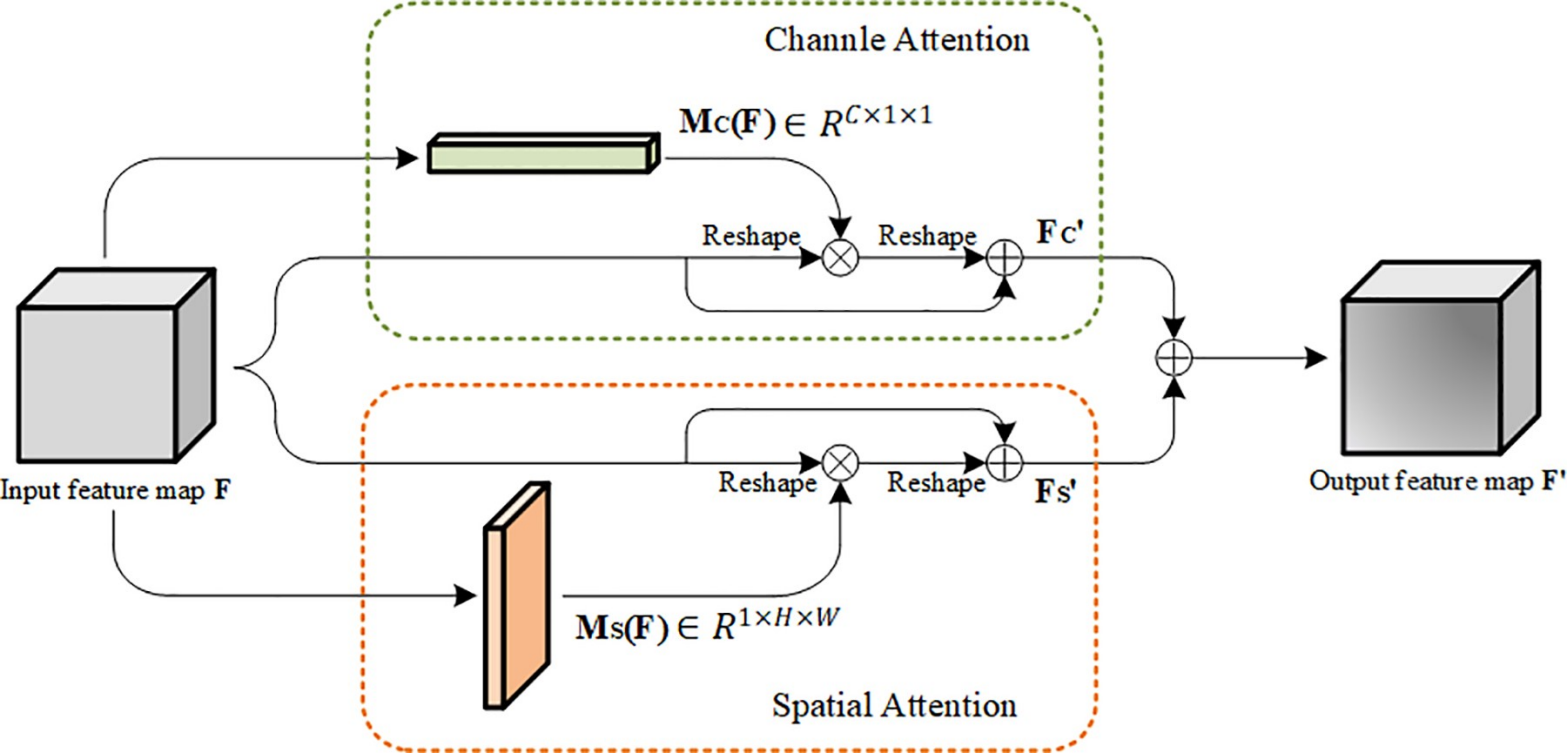
Overall structure of CSAM with parallel channel attention and spatial attention.

Inspired by the work of Woo et al. [31], we constructed a parallel attention module of channel attention and spatial attention, which we called CSAM. Fig 2 shows the overall structure of the CSAM. For input feature maps F∈R^C×H×W^, the module can obtain two attention maps M_C_(F)∈R^C×1×1^ and M_S_(F)∈R^1×H×W^ from the two dimensions of channel and spatial, respectively. Therefore, the channel and spatial feature maps can be expressed as:

$$F_{C}^{\prime }=F+F⊗M_{C}(F)$$ (1)

$$F_{S}^{\prime }=F+F⊗M_{S}(F)$$ (2)

In the formula, $F_{C}^{\prime }$ represents the channel feature map, $F_{S}^{\prime }$ represents the spatial feature map, ⊗ represents multiply by elements. In addition, the residual structure is added to the two attention branches of the model to promote gradient transfer. Finally, the output of the two attention modules is aggregated to extract more effective features. The final output feature map can be expressed as:

$$F\prime =F_{C}^{\prime }+F_{S}^{\prime }$$ (3)

(1) Channel Attention Module

In the input feature map, each channel contains different features of the image, and these features are of different importance to the final classification. So we need to adjust the weight of different channels. The module aggregate the input feature map F in the spatial dimension through max pooling and the average pooling, respectively. And two channel feature vectors $F_{avg}^{C}\in R^{C\times 1\times 1}$ and $F_{max}^{C}\in R^{C\times 1\times 1}$ with the same dimension are obtained. Two new channel feature vectors are obtained by transferring these two channel feature vectors to a multi-layer perceptron (MLP) with only one hidden layer. Finally, the elements of the two channel feature vectors are summed to obtain the channel attention vector M_C_(F)∈R^C×1×1^. In order to reduce the parameters, we set the dimension of the hidden layer to R^C×1×1/r^, where r is a hyper-parameter that represents the reduction rate of the hidden layer. Woo et al. [31] found that when r equals 16, the network can achieve the best performance. So the channel attention vector can be calculated as:

$$\begin{array}{l} M_{C}(F)=\sigma (MLP(AvgPool(F)+MaxPool(F)))\\ \;=\sigma (W_{1}(W_{0}(F_{Avg}^{C})+W_{1}(W_{0}(F_{Max}^{C}))) \end{array}$$ (4)

Where σ is the Sigmoid activation function. W_0_∈R^C/r×C^ and W_1_∈R^C×C/r^ are shared parameters of MLP, and they are shared for the two channel vectors.

(2) Spatial Attention Module

The spatial attention module pays more attention to the spatial position information in the feature map, which can selectively enhance or suppress the weight of the position. Firstly, the module compresses the input feature map F in the channel dimension through max pooling and average pooling, and obtains two two-dimensional compressed feature maps $F_{Avg}^{S}\epsilon R^{1\times H\times W}$ and $F_{Max}^{S}\epsilon R^{1\times H\times W}$. After that, the module connects the two compressed feature maps by a convolution operation, and obtains the spatial attention map **M**_**S**_(**F**). In order to reduce the parameters, the 1×1 convolution operation is first used to reduce the dimension, and replace the 7×7 convolution kernel with 7×1 and 1×7 convolution kernels in the module. The calculation of the spatial attention map can be expressed as follows:

$$\begin{array}{l} M_{S}(F)=\sigma (f(\left[AvgPool(F);MaxPool(F)\right]))\\ \;=\sigma (f([F_{Avg}^{S};F_{Max}^{S}])) \end{array}$$ (5)

Where σ represents the Sigmoid activation function, and *f* represents the three convolution operations of 1×1, 7×1 and 1×7.

#### 2.2.2 Multi-scale attention DenseNet

In order to extract the features of the whole mammogram, we constructed a multi-scale attention DenseNet. The original building block of DenseNet [32] (BN-ReLU-1×1-BN-ReLU-3×3) is replaced by a multi-scale convolution module, and the CSAM is added to the module.

(1) Dense Block

**Fig 3:**
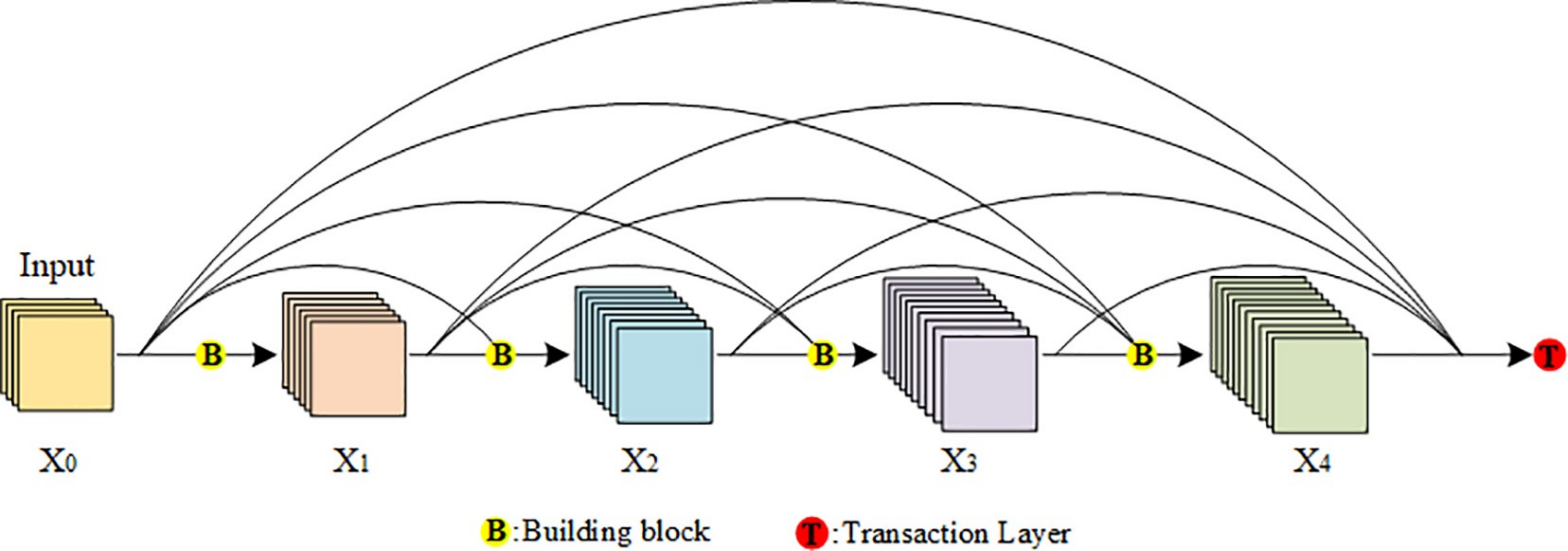
The basic structure of Dense Block. B: Building blocks of Dense Blocks for extracting features of feature map Xi. T: Transaction layer, which is used between two dense blocks to reduce the dimension of output features.

A DenseNet is formed by stacking several Dense Blocks. Fig 3 shows the basic structure of a Dense Block. For Dense Block, in order to achieve feature reuse, each layer will accept all the previous layers as its input. Therefore, for a Dense Block with L layers, it contains L (L+1) / 2 connections. Such a connection can be expressed as:

$$x_{l}=H_{l}(\left[x_{0},x_{1},\cdots ,x_{l-1}\right])$$ (6)

In the formula, H_1_ represents the non-linear conversion function of layer l, which is a combination of operations including batch normalization, ReLU activation function, and convolution. The convolution here refers to the multi-scale convolution we adapt. [x_0_,x_1_,⋯,x_l−1_] represents the connection of feature maps.

Because the feature maps of each layer of the Dense Block are connected to each other, the dimension of the final output feature map will increase significantly. Therefore, a transaction layer is added after the last layer of Dense Block, which is composed of batch normalization layer, 1×1 convolution layer and 2×2 average pooling layer. Through 1×1 convolution, the number of channels of the feature map becomes 1/2 of the original, and through the 2×2 pooling, the size of the feature map becomes 1/2 of the original size.

(2) Multi-scale Convolution Module

**Fig 4:**
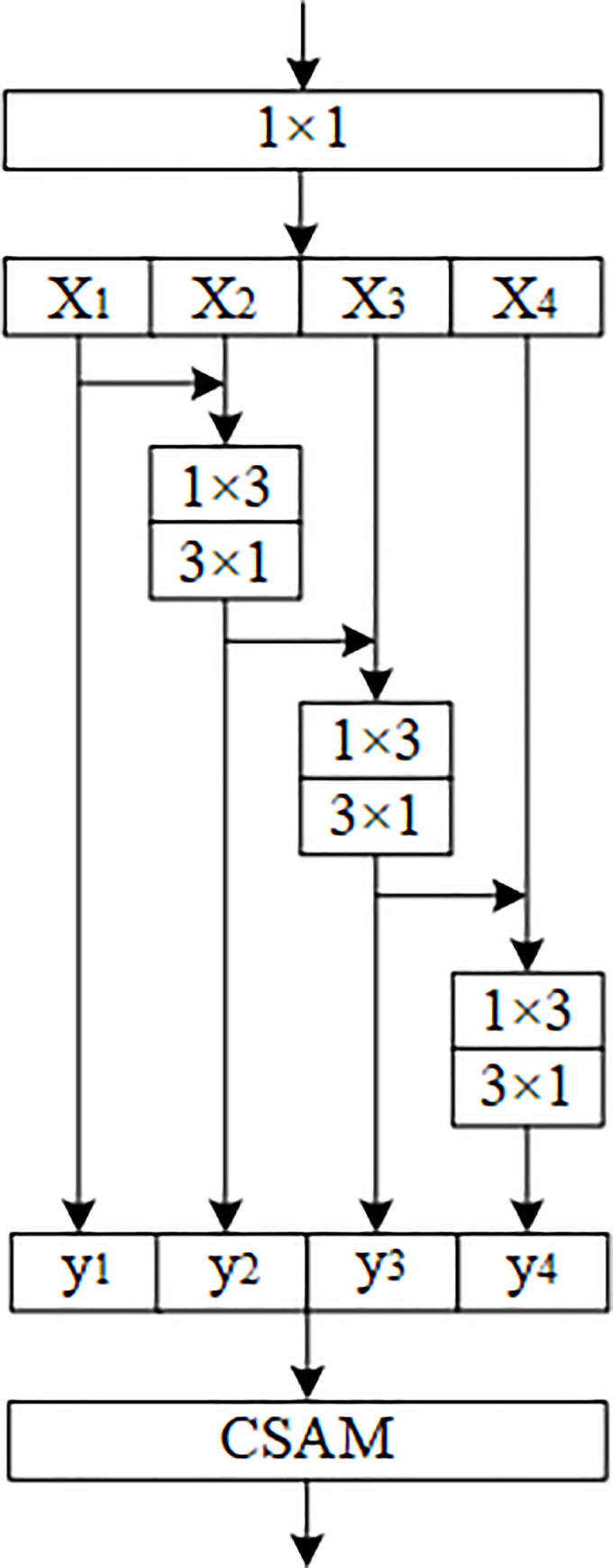
Structure of multi-scale convolution module with CSAM attention module added.

Inspired by the work of Gao et al. [33], we constructed a multi-scale convolution module. The module is used to replace the original building blocks of DenseNet. Fig 4 shows the overall structure of this module. After 1×1 convolution, the channels of the feature map are divided into four parts, which are represented as X_1_, X_2_, X_3_ and X_4_. For the output y_i_ of the feature map subset X_i_, it is stacked with the next feature map subset X_i+1_ to form a new input feature map. Except for the first feature map subset, 3×3 convolution is used to extract the features of different scales of the image layer by layer for the remaining three feature map subsets. Finally, all the outputs y_1_, y_2_, y_3_ and y_4_ are stacked together to form the output feature map of the multi-scale convolution module. Here, a set of 3 × 1 and 1 × 3 convolutions are used instead of a 3×3 convolution. The advantage of this replacement is that it can effectively reduce the number of parameters without affecting the network performance [34]. The output feature map y_i_ can be expressed as:

$$y_{i}=\left\{ \begin{array}{l} X_{i}\;i=1;\\ K_{i}(X_{i}+y_{i-1})\;1<i\leq 4 \end{array}\right.$$ (7)

Where K_i_ represents a set of 3 × 1 and 1 × 3 convolutions. For the i−1th (i≤4) feature subset X_i−1_, its output feature y_i−1_ will be stacked with the ith feature subset x_i_ through the residual structure. The output y_i_ contains the features extracted by X_i−1_ after two sets of 3×1 and 1×3 convolutions. By analogy, for the jth (j>*i*) output feature map y_j_, it contains the features extracted by the feature map subset X_i_ after j−i+1 sets of 3×1 and 1×3 convolutions. After each set of convolutions, the receptive field of feature extraction will expand. Therefore, the output of the module (y_1_, y_2_, y_3_ and y_4_) contains a combination of features of different scales.

In order to enhance the feature expression ability of the network, the attention module CSAM is integrated into the multi-scale convolution module. CSAM can enhance the ability of feature expression from the two dimensions of channel and spatial. Such integration can effectively improve the performance, we have verified in the experiment.

(3) Parameters of multi-scale attention DenseNet

In order to extract the features of whole mammograms, we constructed a multi-scale attention DenseNet. The multi-scale convolution module with CSAM is used as the basic building block to construct a dense network model with a depth of 186. In order to adapt to the network input, the size of the whole mammogram is adjusted to 512 × 512. Table 2 shows the parameters of this multi-scale attention DenseNet.

**Table 2:** Parameters of the multi-scale attention DenseNet with a depth of 186.

Layer	Output Size	Multi-scale DenseNet
Convolution	256×256	7×7 Convolution, stride 2
Pooling	128×128	3×3 Max Pool, stride 2
Dense Block (1)	128×128	CSAM-MS Block × 6
Transaction Layer (1)	128×128	1×1 Convolution
	64×64	2×2 average pool, stride 2
Dense Block (2)	64×64	CSAM-MS Block × 12
Transaction Layer (2)	64×64	1×1 Convolution
	32×32	2×2 average pool, stride 2
Dense Block (3)	32×32	CSAM-MS Block × 24
Transaction Layer (3)	32×32	1×1 Convolution
	16×16	2×2 average pool, stride 2
Dense Block (4)	16×16	CSAM-MS Block × 32
Transaction Layer (4)	16×16	1×1 Convolution
	8×8	2×2 average pool, stride 2
Dense Block (5)	8×8	CSAM-MS Block × 16
Pooling	1×1	8×8 global average pool

CSAM-MS Block represents the multi-scale convolution module with CSAM attention module

#### 2.2.3 Multi-view feature fusion strategy

Mammograms usually need to be acquired from two different views of CC and MLO. In order to make full use of the complementary relationship between the two views, we propose a multi-view feature fusion network model. As shown in Fig 1, the model contains two independent branches. which use the proposed multi-scale attention DenseNet to extract the deep features of mammograms from the CC and MLO views, and each branch can obtain a set of corresponding feature maps. Then a fully connected layer with 1024 hidden nodes is used to connect these two feature maps to form a new feature vector, and finally realize the classification of mammograms. Compared with the features extracted from the single view of the mammogram, the fusion of multi-view features can help to find the internal relationship between different views.

## 3 Experiment and results

In this section, we use the DDSM database to evaluate the performance of the model. We set up three sets of comparative experiments to verify the effectiveness of the proposed model. We analyze their accuracy, sensitivity, area under ROC curve (AUC) and the heat map obtained by visualization of the model. In the last part of this section, we separately evaluate the diagnostic performance of the MVNN model for mass lesions and calcified lesions in two classification tasks, and evaluate the generalization performance of the model through a new test set.

### 3.1 Experimental setting

In this study, the deep learning framework Pytorch [35] is used to build the model. All experiments are conducted on a workstation equipped with four NVIDIA GTX 1080 GPUs with 8G memory, and the workstation uses the Linux operating system. 2853 pairs of two-view mammograms in the DDSM database are used to evaluate the performance of the model, among which 256 pairs of two-view mammograms are only used for model testing. And ten-fold cross-validation is used to evaluate the performance of different models. In the training process of the model, the stochastic gradient descent (SGD) with momentum of 0.9 is used to optimize the network parameters. Based on experience, we set the initial learning rate to 0.001 and the weight attenuation to 1×10^−4^. The mini-batch size is set to 16, because the feature extraction of the whole mammogram requires a lot of memory. The training process iterated 300 epochs.

### 3.2 Training strategy of MVNN

The MVNN model contains two independent branches, and two CNN models with the same structure are used to extract the features of mammograms from CC and MLO views, respectively. Then the last convolutional layer of these two feature extraction networks are connected through a fully connected layer with 1024 nodes. We trained the model according to the experimental settings in section 3.1. First, two feature extraction networks in MVNN are trained by the enhanced single view mammography image, and their convolution layer parameters are fixed. After that, the enhanced two-view mammograms are used as input of the MVNN model to fine-tune the fully connected layers, and finally achieved the classification of the two-view mammograms. It should be noted that the parameters of the convolutional layer were not updated in the second step.

### 3.3 Comparative experiment

#### 3.3.1 Influence of multi-view feature fusion strategy on classification performance

**Fig 5:**
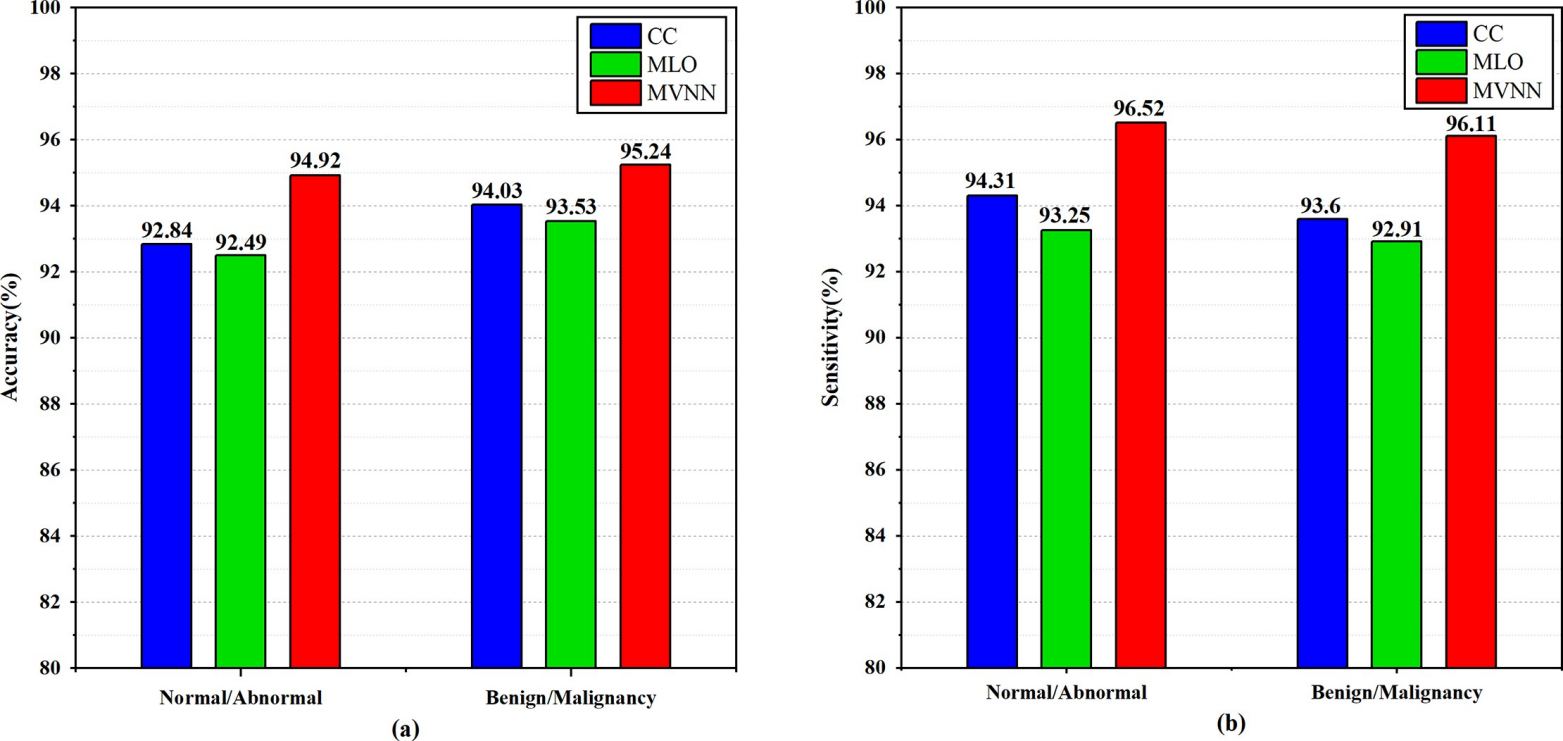
The performance comparison between two single-view networks and the proposed MVNN in the two-stage classification tasks. (a) Accuracy, (b) Sensitivity.

For radiologists, they usually need to combine the mammograms from CC and MLO views to make judgments, and MVNN is inspired by this. Compared with the single-view mammograms, the two-view mammograms contain richer information. If this information can be fully utilized, it will help improve the classification performance of the model. Therefore, in order to verify the effectiveness of the two-view mammograms feature fusion, we set up a set of comparative experiments. Two multi-scale attention DenseNets are used to classify mammograms from CC and MLO views, respectively. Their accuracy and sensitivity in two tasks are shown in Fig 5.

The experimental results show that, compared with the MLO, diagnosis through the CC view can obtain more accurate results. At the same time, the accuracy and sensitivity of the MVNN model in both classification tasks are higher than the single-view classification network. Therefore, it can be proved that the classification performance can be effectively improved by fusing features from different views.

#### 3.3.2 Influence of multi-scale convolution module on classification performance

In this study, in order to effectively extract the features of lesions, we proposed a multi-scale convolution module and apply it to the DenseNet. In this section, a set of comparative experiments are used to verify the effectiveness of the proposed multi-scale convolution module. The proposed multi-scale convolution module is compared with the original Dense Block [32] and two currently popular multi-scale convolution modules [36, 37], respectively. In order to integrate these two multi-scale convolution modules into DenseNet, they are fine-tuned. And CSAM is integrated into all the modules used in the experiment to control the variables.

**Fig 6:**
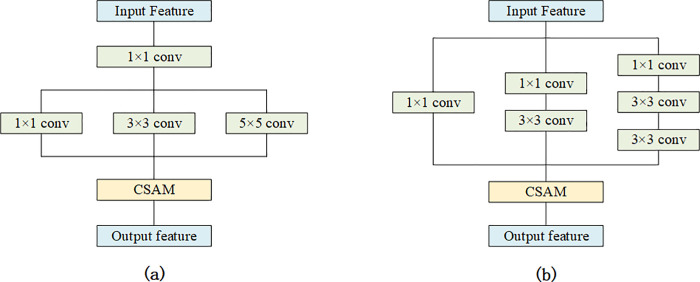
Two popular multi-scale convolution modules. (a) An ordinary multi-scale convolution module, (b) multi-scale convolution module inspired by Inception structure.

Fig 6 shows the structure of the two multi-scale convolution modules used in the experiments. Fig 6A is a simple multi-scale convolution module proposed by Moya et al. [36]. After 1×1 convolution, three convolution kernels with different scales are used to extract image features. Firstly, a 1 × 1 convolution layer is used to reduce the dimension of the input feature map, and then three convolution kernels with different scales are used to extract the image features of different scales. Fig 6B is proposed by Chen et al. [37], and their work was inspired by the Inception structure. The module contains three branches, which extract image features at different scales. And in each branch, a 1×1 convolution operation is first used to reduce the dimension of the feature map. Compared with the module in Fig 6A, this module can effectively reduce the number of parameters.

**Fig 7:**
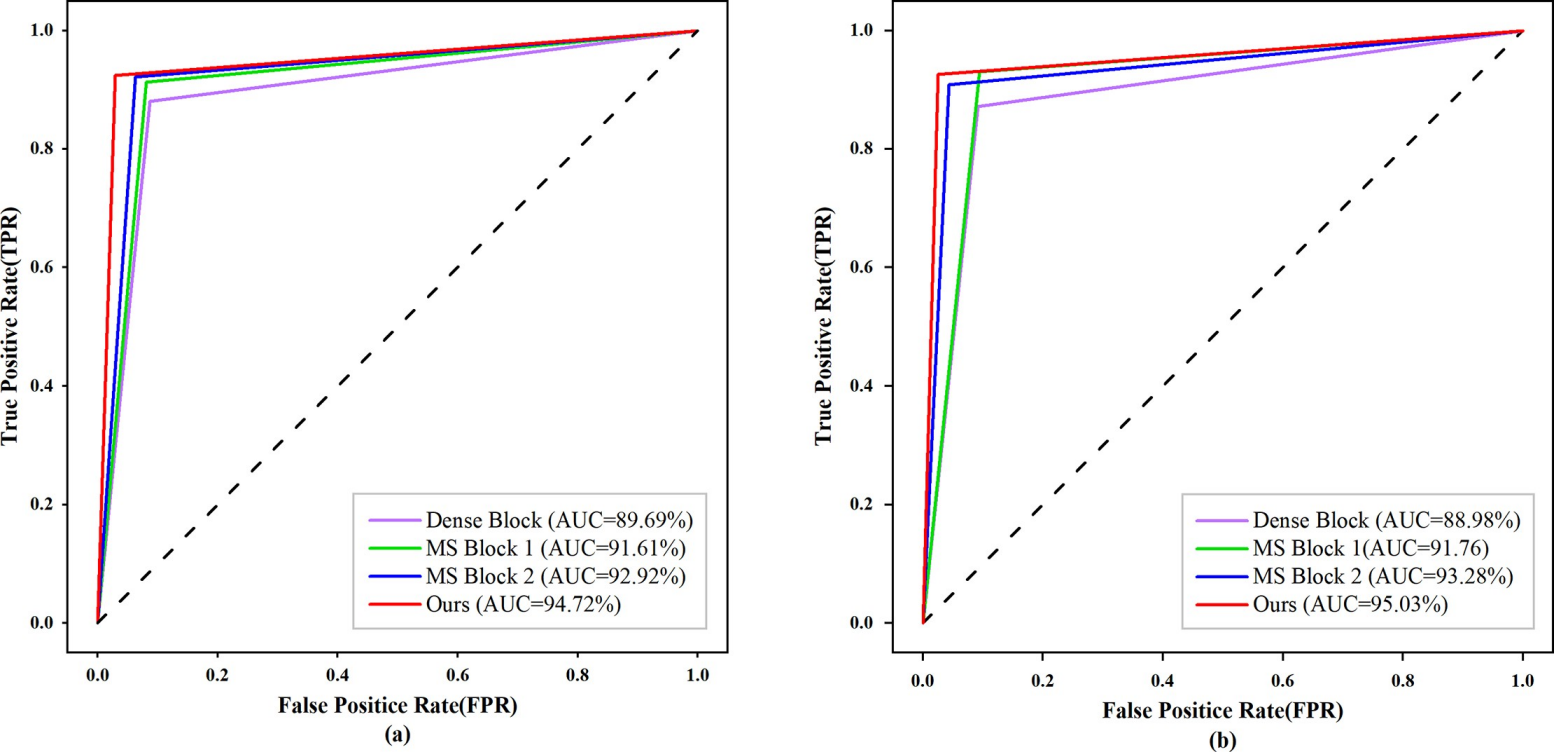
ROC curves of different multi-scale convolution modules and original DenseNet. (a) ROC curve of normal and abnormal classification, (b)ROC curve of Benign and Malignancy classification.

In this set of experiments, the multi-scale convolution module in MVNN is replaced by the three modules mentioned before, and the experiments are conducted according to the requirements of Sections 3.1 and 3.2. Table 3 shows the classification accuracy and sensitivity of MVNN with different convolution modules, and their receiver operating characteristic (ROC) curves in two classification tasks are shown in Fig 7.

**Table 3:** Parameters of the multi-scale attention DenseNet with a depth of 186.

Building Block	Classification	Accuracy	Sensitivity
**Dense Block [27]**	Normal/Abnormal	91.49%	92.11%
	Benign/Malignancy	90.97%	91.79%
**Multi-scale Block 1 [28]**	Normal/Abnormal	92.14%	93.24%
	Benign/Malignancy	92.32%	92.21%
**Multi-scale Block 2 [29]**	Normal/Abnormal	93.76%	94.67%
	Benign/Malignancy	94.46%	94.58%
**Proposed**	Normal/Abnormal	**94.92%**	**96.52%**
	Benign/Malignancy	**95.24%**	**96.11%**

**Fig 8:**
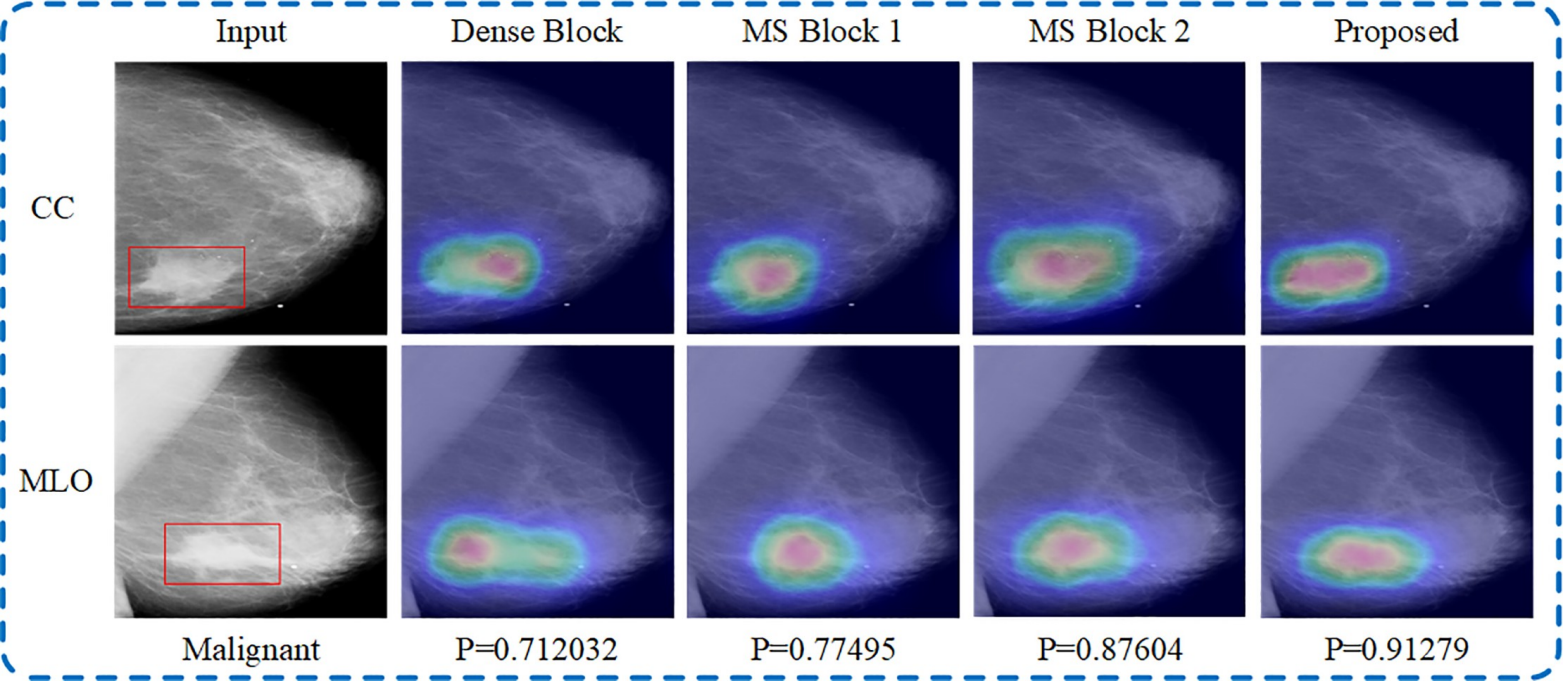
Visualization results of the model with different convolution module in normal and abnormal classification task. This case is malignant, and P denotes the corresponding softmax score in the benign and malignant classification task.

It can be seen from the experimental results that the accuracy, sensitivity and AUC of the multi-scale convolution module we proposed are higher than other modules. The main reasons for the good performance of this module are as follows: First, the residual structure is used in the multi-scale convolution module, which can increase the range of receptive field layer by layer, and extract the features of different scales more accurately. Second, the module uses 1×1 convolution to reduce the dimension of the feature map, and replaces 3×3 convolution with a combined convolutional layer of 3×1 and 1×3, which can effectively reduce the number of parameters. The model in the normal and abnormal classification task is visualized to prove that it can focus on the lesion area more accurately. Fig 8 shows the visualization results of the model, and the last line of the figure gives the probability that the case is malignant. Each of the input mammograms from the CC and MLO views contains a mass lesion. It can be seen from the change trend of the four sets of heat maps that our proposed multi-scale convolution module can focus on the lesion area more accurately. And from the change trend of probability, it can be seen that the model can provide more reliable classification results of benign and malignant.

#### 3.3.3 Influence of attention module on classification

**Fig 9:**
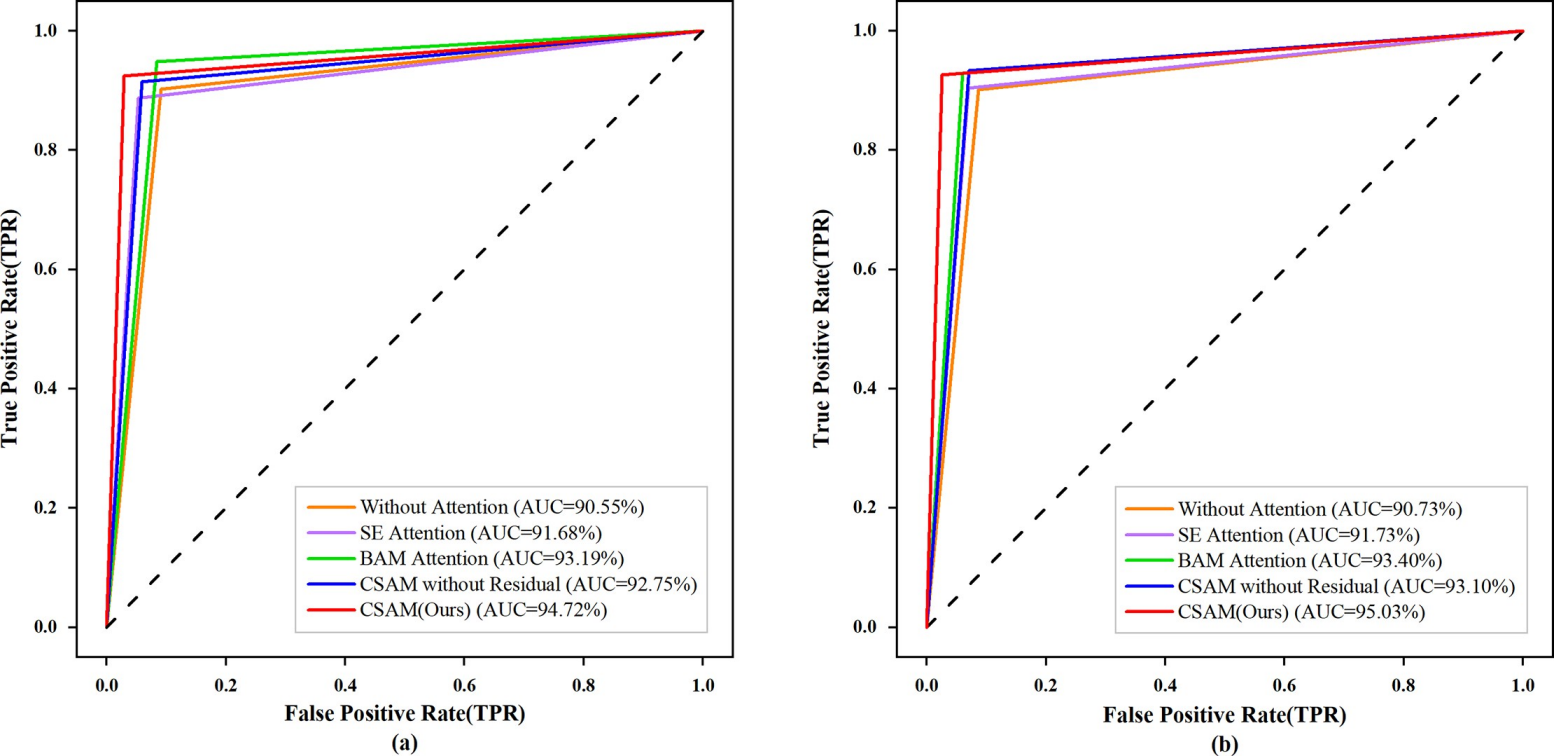
ROC curves of different attention modules in two-stage classification tasks. (a) Normal and Abnormal classification, (b) Benign and Malignancy Classification.

In this set of experiments, the MVNN model integrates different attention modules to verify the effectiveness of CSAM. Firstly, a multi-scale DenseNet without attention is used to evaluate the effect of attention module on model performance. Secondly, two popular attention modules, squeeze and exception (SE) module [38] and bottleneck attention module (BAM) [39] are integrated into the model to compare with the performance of CSAM. SE module is a channel attention module proposed by Hu et al. [38]. The advantage of this module is that it contains very few parameters and can selectively highlight useful features in channel dimension. The BAM is proposed by Park et al. [39]. This module constructs a 3D attention map through two parallel paths of channel and spatial. However, the module contains several convolution layers which lead to a large number of parameters, so it can only be used in the bottleneck layer of the network. Finally, because the residual structure is used in the CSAM module, we set up a CSAM module without the residual structure to verify the effect of the residual structure on the module performance. The accuracy and sensitivity of these models in the two classification tasks are shown in Table 4, and their ROC curves are shown in Fig 9.

**Table 4:** Influence of different attention modules on model classification performance.

Attention	Classification	Accuracy	Sensitivity
**Without Attention**	Normal/Abnormal	91.41%	91.68%
	Benign/Malignancy	91.90%	91.93%
**SE** [38] **Attention**	Normal/Abnormal	92.68%	93.18%
	Benign/Malignancy	93.11%	93.05%
**BAM** [39] **Attention**	Normal/Abnormal	94.03%	95.02%
	Benign/Malignancy	94.31%	94.71%
**CSAM Without Residual**	Normal/Abnormal	93.92%	95.45%
	Benign/Malignancy	94.03%	94.99%
**CSAM (Ours)**	Normal/Abnormal	**94.92%**	**96.52%**
	Benign/Malignancy	**95.24%**	**96.11%**

**Fig 10:**
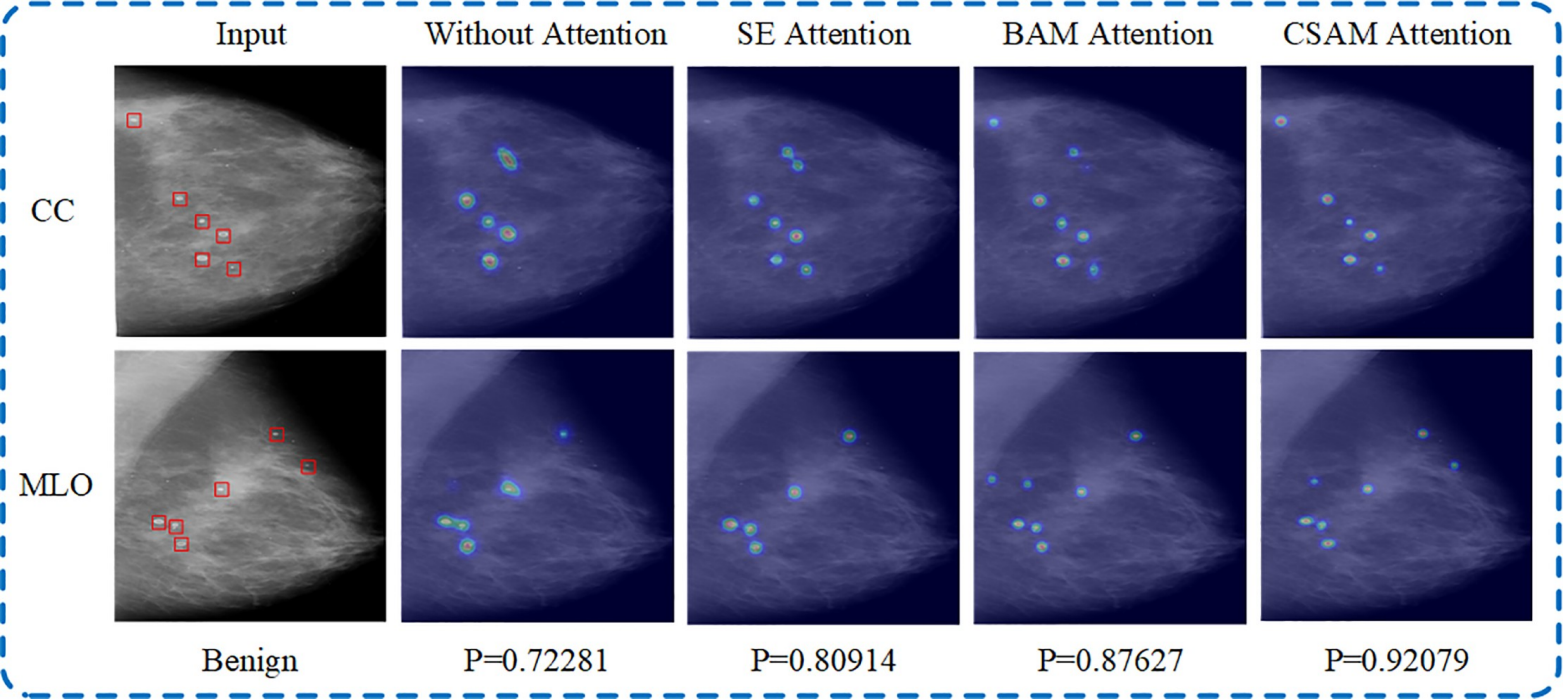
Visualization results of the model with different attention module in normal and abnormal classification task. This case is benign, and P denotes the corresponding softmax score in the benign and malignant classification task.

It can be seen from the experimental results that the use of the attention module can effectively improve the classification performance of the model. And compared with other attention modules, the MVNN model integrated with CSAM has higher accuracy, sensitivity and AUC in both classification tasks. The advantages of CSAM are as follows: First, this module can enhance the expression ability of features from the two dimensions of channel and spatial at the same time. Second the number of parameters included in this module is small, which will not seriously increase the computational burden of the model. In addition, the models with different attention modules in the normal and abnormal classification task are visualized to prove that the CSAM can help the model focus on the important information. Fig 10 shows their visualization results, and the last line of the figure gives the probability that the case is benign. Each of the original two-view mammograms contains 6 calcification points. It can be seen from the change trend of four sets of heat maps that the MVNN model with CSAM more accurately focuses on the lesion area, and also has better identification ability for small lesions and non-lesion areas. And in the benign and malignant classification tasks, CSAM can also help the model provide more reliable probability.

### 3.4 Model analysis and testing

For abnormal mammograms, there are two types of lesions: mass and calcification. In order to comprehensively evaluate the performance of the MVNN model, we obtained the diagnostic accuracy of the two lesions in the two classification tasks through ten-fold cross-validation. Our training set contains a total of 1407 abnormal breasts, of which 788 breasts contain mass lesions (387 benign and 401 malignant), and 619 breasts contain calcified lesions (301 benign and 318 malignant). For the classification tasks of normal and abnormal mammograms, we evaluated the accuracy of the mass and calcified lesions correctly classified as abnormal mammograms. For the classification task of benign and malignant mammograms, we separately evaluate the accuracy of two different lesions. The diagnostic accuracy of these two lesions in the two classification tasks are shown in Table 5. Compared with calcified lesions, mass lesions are often more obvious, so a higher accuracy rate is obtained.

**Table 5:** Diagnostic accuracy of mass lesions and calcified lesions in two classification tasks.

Type	Mass	Calcification
**Normal/Abnormal**	97.08%	95.80%
**Benign/Malignancy**	95.43%	94.99%

In addition, in order to evaluate the generalization performance of the model, 256 pairs of two-view mammograms in the DDSM database are used to test the model. Among them, 128 pairs are normal, 64 pairs are benign and 64 pairs are malignant. The model does not use these mammograms in the process of training. We used the trained MVNN model to classify these mammograms. Table 6 shows the test results of the model. Experimental results show that the MVNN model has strong robustness and can effectively classify new data.

**Table 6:** Test performance of the MVNN model.

Classification	Accuracy	Sensitivity
**Normal/Abnormal**	95.31%	96.09%
**Benign/Malignancy**	96.09%	96.88%

## 4 Discussion

### 4.1 Advantage of the proposed method

In this paper, we proposed a multi-view feature fusion network model to classify mammograms, and the model has achieved good classification performance in two-stage classification tasks. The visualization results of the model show that the model has the ability to roughly locate the lesion area, which can help the radiologist to quickly find the lesion. And in the classification task of benign and malignant, the model can provide a more reliable diagnosis result of benign and malignant to the radiologist for reference. The main reasons for the good performance of this model are as follows: Firstly, the model contains two CNN branches, which extract the features of mammograms from two views, so that the network can focus on a wider range of spatial information. Secondly, the proposed multi-scale convolution module enables the network to extract the features of different scales in the image. Finally, the proposed attention module CSAM can help the model to selectively focus on useful information.

### 4.2 Compare with state-of-the-art

The advantage of deep learning is that it can extract deep features from images that people cannot notice. Table 7 shows the performance comparison between our method and other deep learning based methods. They include the classification of whole mammograms and multi-view image patches.

**Table 7:** Compare with State-of-the-art.

	Classification	Accuracy	Sensitivity	AUC
**Shu et al. [40]**	Benign/Malignancy	92.2%	--	92.4%
**Shams et al. [41]**	Benign/Malignancy	93.5%	--	92.5%
**Li et al. [42]**	Benign/Malignancy	94.55%	95.60%	91.2%
**Sun et al. [43]**	Benign/Malignancy	82.02%	--	--
**Khan et al. [23]**	Normal/Abnormal	93.73%	96.31%	93.4%
	Benign/Malignancy	77.66%	81.82%	76.9%
**Ours**	Normal/Abnormal	94.92%	96.52%	94.72%
	Benign/Malignancy	95.24%	96.11%	95.03%

(1) Shu et al. [40] proposed a classification method of whole mammograms based on deep neural network. In order to solve the problem of classification of small lesions in the whole mammogram, they proposed two structures: region max pooling and global maximum pooling. They used a pre-trained AlexNet for benign and malignant classification, and applied the proposed two pooling structures to the network. Shams et al. [41] constructed a classification network for whole mammograms, which consists of a feature extraction network and an extended classification network. They trained the network using patches and whole mammograms, and used the DDSM database for pre-training. Li et al. [42] proposed a DenseNet-II model for benign and malignant classification of whole mammograms. They added an Inception structure in front of the DenseNet model to extract the multi-scale features.

(2) Sun et al. [43] proposed an MVMDCNN for benign and malignant classification of two-view mammogram patches. The model combines multi-view CNN and multi-detail CNN. Firstly, multi-view CNN is used to fuse two-view image features, and then multi-detail CNN is used to extract deep features. Khan et al. [23] constructed a four-view classification network, which divides mammogram patches into normal and abnormal, mass and calcification, benign and malignant in three stages.

According to the comparison results in Table 7, we can see that whether it is classification of normal and abnormal or classification of benign and malignant, our method has better performance. And it can also be found that whether it is the multi-scale CNN or the multi-view CNN, the purpose of the researchers is to extract more useful features to improve the classification performance. Our model combines multi-view CNN and multi-scale CNN, and compared with the above research, the performance of our model has been improved.

### 4.3 Limitation and future work

Although our research has improved the classification performance of mammograms, there are still some problems to be solved. Firstly, this study uses different data enhancement strategies to expand the training data, but the model performance is still limited by the amount of data. Therefore, in future work, we hope to improve the performance of the model through transfer learning based on similar data. Secondly, our research focuses on verifying the effectiveness of the proposed multi-scale convolution module and attention module, but there is not much discussion on the feature fusion method. In the future work, we will explore a more effective feature fusion strategy. Due to the limitation of time, we will solve these problems in the future work, and we believe that these work will help improve model performance.

## 5 Conclusion

In this study, we proposed a multi-view feature fusion network model to classify two-view mammograms in two stages. In the first stage, mammograms are divided into normal and abnormal, and in the second stage, the abnormal mammograms are divided into benign and malignant. Our work mainly focuses on the construction of multi-scale convolution module and attention module. Firstly, the multi-scale convolution module uses different scale convolution kernels to extract image features, so the network can extract the information of the image at different scales. Secondly, the attention module we proposed contains two independent branches of channel attention and spatial attention, which can enhance the ability of feature expression in channel and spatial dimensions. Finally, the DDSM database is used to verify the proposed method, and has achieved good experimental results. Compared with the current research, our work has made improvement.
